# Assessment of non-tumor liver parenchyma damage in advanced gastric cancer treatment with transarterial infusion chemotherapy: a study using imaging and hepatic injury indicators

**DOI:** 10.3389/fonc.2025.1537688

**Published:** 2025-02-19

**Authors:** Yifan Jing, Jian Jing, Xiaokang Sun, Yong Jin, Xuming Bai

**Affiliations:** Interventional Therapy Department, The Second Affiliated Hospital of Soochow University, Suzhou, China

**Keywords:** transarterial infusion chemotherapy, non-tumor liver parenchyma damage, CT imaging, hepatic injury indicators, advanced gastric cancer

## Abstract

**Introduction:**

This study aimed to evaluate non-tumor liver parenchymal injury in advanced gastric cancer patients undergoing transarterial infusion chemotherapy (TAI) using imaging parameters and liver injury biomarkers, providing objective evidence for early detection of drug-induced liver injury.

**Methods:**

A retrospective analysis was conducted on 52 advanced gastric cancer patients who received TAI at our center from July 2015 to July 2023. Abdominal CT images and laboratory data were collected before and after treatment. Imaging postprocessing software was used to measure the liver-to-spleen (L/S) attenuation ratio and spleen volume. Liver fibrosis indices (APRI, FIB-4) and liver function scores (Child-Pugh, ALBI) were calculated. Statistical analysis included Wilcoxon rank-sum test and paired t-test for pre- and post-treatment comparisons.

**Results:**

Patients received an average of 2.80 TAI cycles over 8.3 weeks. Post-treatment, 76.92% (40/52) showed a significant reduction in L/S attenuation ratio (1.23 ± 0.13 vs. 1.12 ± 0.1, P < 0.01), and 73.1% (38/52) exhibited increased spleen volume (151,219.33 mm^³^ vs. 202, 171.32 mm^³^, P < 0.01). Liver fibrosis indices significantly increased: APRI (0.19 ± 0.15 vs. 0.37 ± 0.27) and FIB-4 (1.29 ± 0.88 vs. 2.24 ± 1.38) (P < 0.01). No significant changes were observed in ALBI (-2.7 ± 0.41 vs. -2.58 ± 0.43) or Child-Pugh scores (5.31 ± 0.47 vs. 5.38 ± 0.64) (P > 0.05).

**Discussion:**

Transarterial infusion chemotherapy for advanced gastric cancer results in short-term damage to the non-tumor liver parenchyma, with imaging findings showing hepatic steatosis (reduction in L/S ratio) and splenomegaly. Serum markers suggest progression of hepatic fibrosis, consistent with the pathological features of hepatic sinusoidal obstruction syndrome. The combination of CT imaging and APRI/FIB-4 provides a sensitive method for detecting subclinical liver injury, reflecting pathological changes earlier than traditional liver function scores.

## 1 Introduction

Gastric cancer is the fifth most common cancer worldwide and one of the three leading causes of cancer deaths (1). In China, early gastric cancer accounts for approximately 20% of all gastric cancers, and the majority of gastric cancer patients are already in advanced stages when they seek treatment, resulting in a 5-year survival rate of less than 50% (2). Chemotherapy is a common treatment for advanced gastric cancer, and improving its efficacy, especially with neoadjuvant chemotherapy and translational therapy, thus increasing the rate of surgical resection, is an important factor affecting patient survival. In recent years, neoadjuvant chemotherapy has been gradually applied in the treatment of advanced gastric cancer and has achieved excellent clinical efficacy (3). Neoadjuvant chemotherapy is effective in reducing the tumor lesion size, lowering the tumor stage, and increasing the rate of radical treatment (4). Early systemic therapy also provides rapid relief from symptoms, eliminates tumor micrometastases, and prevents intraoperative tumor spread and postoperative tumor recurrence.

Recently, with advancement of minimally invasive techniques, such as endovascular intervention, transarterial infusion chemotherapy has emerged as a new neoadjuvant chemotherapy method, which allows direct delivery of chemotherapy drugs into the target organ through blood supply arteries, increasing drug concentration in the tumor area and enhancing the therapeutic effect. Compared to intravenous infusion, transarterial infusion chemotherapy significantly increases local tumor drug concentrations and improves efficacy (5). Arterial infusion has a higher tumor response rate than that of intravenous infusion. FOLFOX regimen chemotherapy for progressive hepatocellular carcinoma has a tumor response rate of 8.15% for intravenous infusion compared to a 46% tumor response rate for arterial infusion (6, 7). In chemotherapy for colorectal cancer liver metastases, the tumor objective response rate to transarterial infusion of fluorouracil analogues was significantly higher than that to intravenous infusion (41 vs. 14%; P < 0.01) (8). The conventional transarterial infusion chemotherapy is most commonly used in liver cancer, and its use in hollow organ is rare. In recent years, with the advancement of interventional techniques, transarterial chemotherapy infusion has achieved promising results in the treatment of cancers of hollow organs such as gastric, colorectal, bladder, and ovarian cancers (9–11), with particularly significant effects observed in gastric cancer (12–16). Therefore, it is recommended in many current clinical guidelines for tumor treatment (17–19).

However, the adverse effects of transarterial infusion chemotherapy, especially the damage caused by high concentrations of chemotherapeutic agents to adjacent normal tissues and organs, have rarely been reported and are only briefly mentioned or summarized in the relevant literature as undetailed hepatotoxicity (8). Therefore, to address this phenomenon, this study investigated the impact of transarterial infusion chemotherapy on non-tumor liver parenchyma in 52 patients with advanced gastric cancer.

## 2 Materials and methods

This single-center, retrospective, observational study was reviewed and approved by the institutional review board(JD-HG-2024-244). The requirement for informed consent was waived because this study was based on routinely collected claims data.

### 2.1 Clinical data

We retrospectively analyzed the data of 60 patients with locally advanced gastric cancer who received transarterial infusion chemotherapy in the interventional unit of our hospital between July 2015 and July 2023. Inclusion criteria: complete preoperative and postoperative plain CT and enhanced CT of the upper abdomen, complete relevant laboratory parameters, ECOG (physical status) score ≤ 1. Patients undergo a minimum number of infusion treatment cycles greater than or equal to 2. Exclusion criteria: patients involved in other therapeutic regimens, patients with a combination of other malignant tumors, patients with severe underlying liver disease such as various types of cirrhosis, viral hepatitis, and moderate-to-severe fatty liver. Fifty-two patients who met the criteria were screened. The baseline and tumor characteristics of the patients are presented in **Table 1**.

**Table 1:** Baseline and tumor characteristics of the patients.

	Patients	Proportions
Age (years)		
≥60	38	0.73
<60	14	0.27
Sex		
Male	32	0.62
Female	20	0.38
Histology		
Poorly differentiated	24	0.46
Moderately poorly differentiated	20	0.38
Mucinous Carcinoma	6	0.12
Signet ring cell carcinoma	2	0.04
ypTNM stage		
IIIa	20	0.38
IIIb	18	0.35
IIIc	14	0.27
Cycle of treatment (Times)		
3	40	0.77
2	12	0.23

### 2.2 Transarterial infusion chemotherapy

Chemotherapy regimen: oxaliplatin 100 mg/m ², etoposide 80 mg/m ², epimedium 30 mg/m ², and S-1 40–60 mg/m ².

Procedure: The 4F vascular sheath was inserted percutaneously into the femoral artery using the modified Seldinger technique. The RH catheter was then directed into the celiac axis and connected to an external high-pressure injector for high-pressure angiography. Based on the lesion location and the main responsible vessel, the microcatheter was advanced into the main vessel and left in place. The retention locations of the microcatheter are shown in **Table 1**.

Medication methods: On the first day, half of the chemotherapeutic drugs were infused via a microcatheter in the following order: oxaliplatin, etoposide, and epothilone; each drug was infused for 1 h. On the second day, the microcatheter was retreated to a 4F RH tube in the celiac trunk, and the remaining drugs were infused in the celiac axis in the same order and at the same time as before. Additionally, all patients received oral S-1, (40–60 mg, twice a day; the total dose depending on the patient’s body surface area as follows: <1.25 m^2^, 80 mg; 1.25–1.50 m^2^, 100 mg; and >1.50 m^2^, 120 mg; on days 1–14 of every 3-week cycle. Treatment cycles were performed every 3 weeks. Imaging and laboratory tests were reviewed after 2-3 cycles for evaluation.

### 2.3 Evaluation indicators

#### 2.3.1 Imaging evaluation indicators

**Figure 1:**
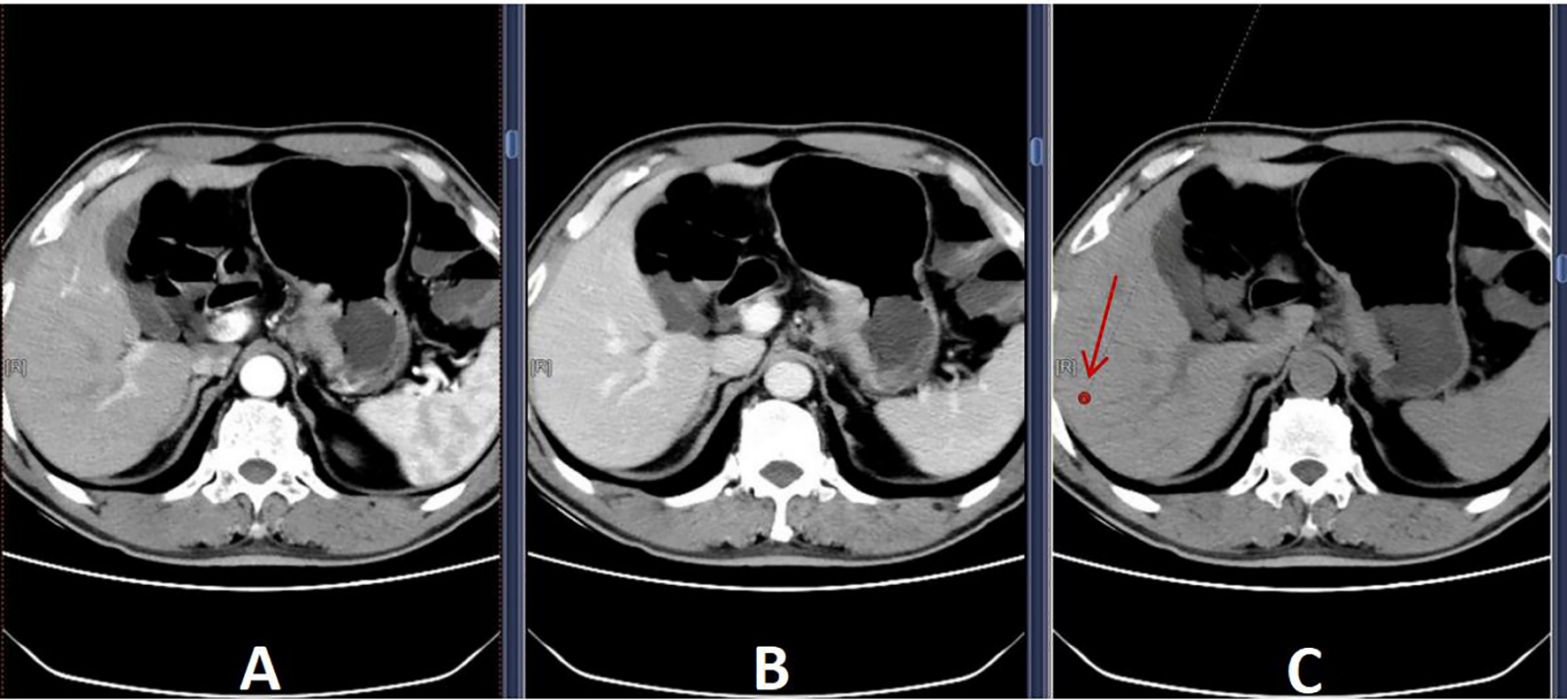
Selected ROIs **(A)** Arterial phase; **(B)** Delayed phase; **(C)** Unenhanced phase. To avoid blood vessels, bile ducts, and areas of heterogeneity, a 1 mm² region-of-interest (arrow) was selected in **(C)** to measure liver density.

1. L/S density ratio measurement and calculation: Hepatic and splenic ratios were measured in the unenhanced phase of CT scans using region-of-interest (ROI) cursors with an area of 1 mm² in the liver and spleen. The ROIs were located in the caudate, left, and right lobes of the liver. In the spleen, ROIs were located at the same level as the selected liver, and all measurements were manually obtained in regions of uniform parenchymal attenuation, with care taken to avoid vessels, artefacts, and other areas that might have spuriously increased or decreased the measurements. The average of the measurements of each segment of the liver and spleen was taken, and the L/S density ratio was calculated (**Figure 1**).
2. Splenic volume measurement: We collected all patients’ upper abdominal CT images in DICOM format and imported thin-layer plain CT images into the 3D-Slicer software (www.slicer.org). Two experienced radiologists identified the optimal levels for outlining the spleen’s ROIs. They traced the spleen at multiple levels, automatically generating 3D stereo images through level tracing and slice filling functions, finally calculating the spleen volume. (To ensure accuracy of the data, the shape and edges of the spleen were carefully observed during the sketching process).

#### 2.3.2 Hepatic fibrosis index

1) APRI(aspartate aminotransferase-platelet ratio index)=


$$\frac{AST\left(/ULN\right)\times 100}{PLT(10^{9}/L)}$$

(20).

2) FIB-4(fibrosis index based on 4 factor)=


$$\frac{age(years)\times AST\left(IU/L\right)}{PLT\;(10^{9}/L)\times \sqrt{ALT\left(IU/L\right)}}$$

(20)

#### 2.3.3 Liver function assessment indicators

1) Liver functional enzyme profiles: ALT, AST, ALP, GGT and TBIL.

2) Albumin-Bilirubin Grade (ALBI)=


$$\log_{10}TBIL(\mu mol/L)\times 0.66+ALB(g/L)\times -0.085$$

(21)

3) Child-Turcotte-Pugh score (22).

### 2.4 Statistical analysis

Statistical analysis was performed using SPSS 27, and the measurement data are expressed as mean $\bar{X}$ ± *S* standard deviation. Data that conformed to normal distribution with chi-square were tested using paired t-test, and data that did not conform to normal distribution were tested using Wilcoxon signed rank sum test. To control the false discovery rate (FDR) in multiple comparisons, we used the Benjamini-Hochberg procedure. All P values were first ranked and then adjusted by the FDR method to control for FDR at the 0.05 level.

## 3 Results

### 3.1 Intervention operations

**Figure 2:**
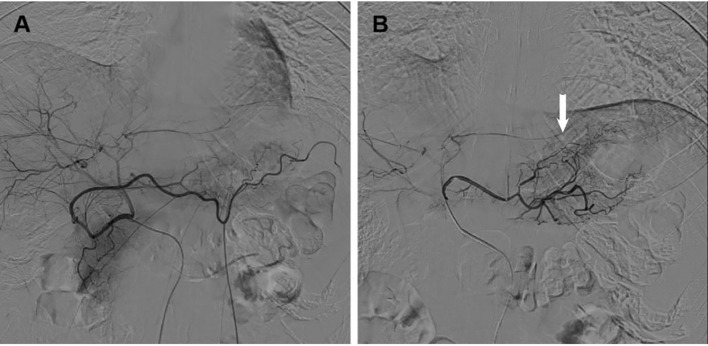
X-ray images of catheter retention positions **(A)** Angiography of the RH catheter in the celiac axis; **(B)** Angiography of microcatheter in tumor blood supplying arteries (Arrow: Tumor blood supplying arteries).

The success rate of the interventional procedure was 100%, with no complications related to the surgical procedure. Catheter retention positions were as follows: RH catheter was left in the celiac axis in 52 cases; The placement of microcatheters are determined based on angiography (details in **Table 1**). The average number of transarterial infusion chemotherapy cycles was 2.77 (X-ray images are shown in **Figure 2**).

### 3.2 Liver/spleen density ratio

L/S density ratio decreased after transarterial infusion chemotherapy in 76.92% patients (40/52), of which 46.15% (24/52) showed decreased postoperative L/S density ratio (to ≤ 1.1). Compared with pre-treatment, the mean reduction in L/S density ratio after treatment was 0.11. After applying the Benjamini-Hochberg procedure for controlling the false discovery rate, the statistically significant difference persisted (1.23 ± 0.13 vs 1.12 ± 0.1, corrected P < 0.01).

### 3.3 Splenic volume

**Figure 3:**
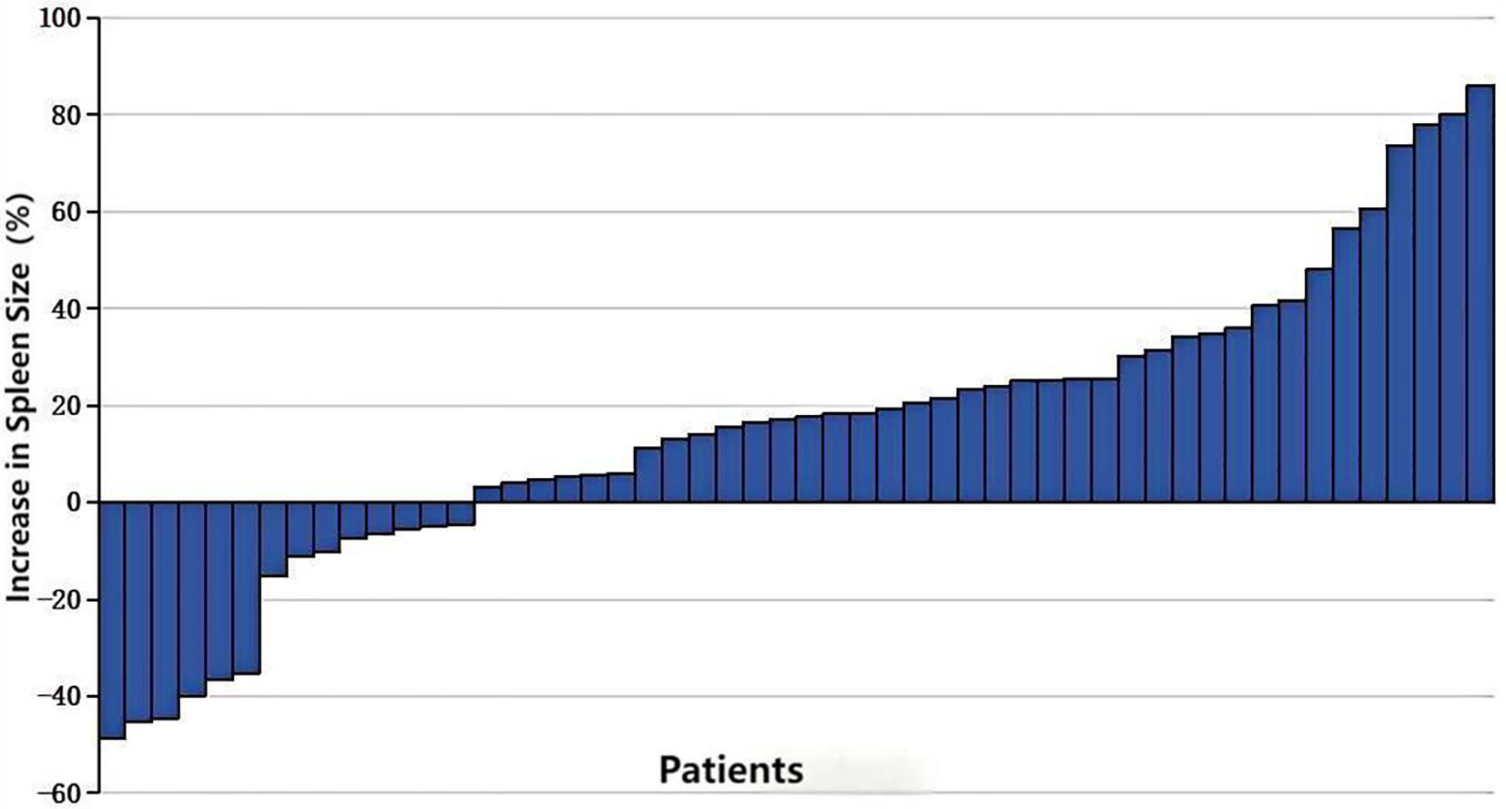
Percentage increase in splenic volume size in patients receiving 2-3 cycles of transarterial infusion chemotherapy.

Following transarterial infusion chemotherapy, spleen volume increased in 73.07% of patients (38/52), with 14 patients showing an increase of more than 30%, and 6 patients experiencing an increase of more than 50% (**Figure 3**). The mean increase in volume was 14% (range: -48.63%–77.78%). The median increase in spleen volume compared to pre-treatment was 50,951.97 mm³ (P < 0.05) (**Table 2**).

**Table 2:** Splenic volume.

	M (P_25_, P_75_)		*z*	*P*
	Pre-operative	Post-operative		
Splenic volume (mm³)	151219.33(113531.32,198856.62)	202171.32(133729.11,247383.23)	2.38	0.02*

* *p*<0.05.

Changes in splenic volume from preoperative to postoperative period.

The Wilcoxon signed rank-sum test showed a statistically significant difference in the postoperative spleen volume compared to the preoperative period (P < 0.05).

### 3.4 Liver fibrosis index

**Figure 4:**
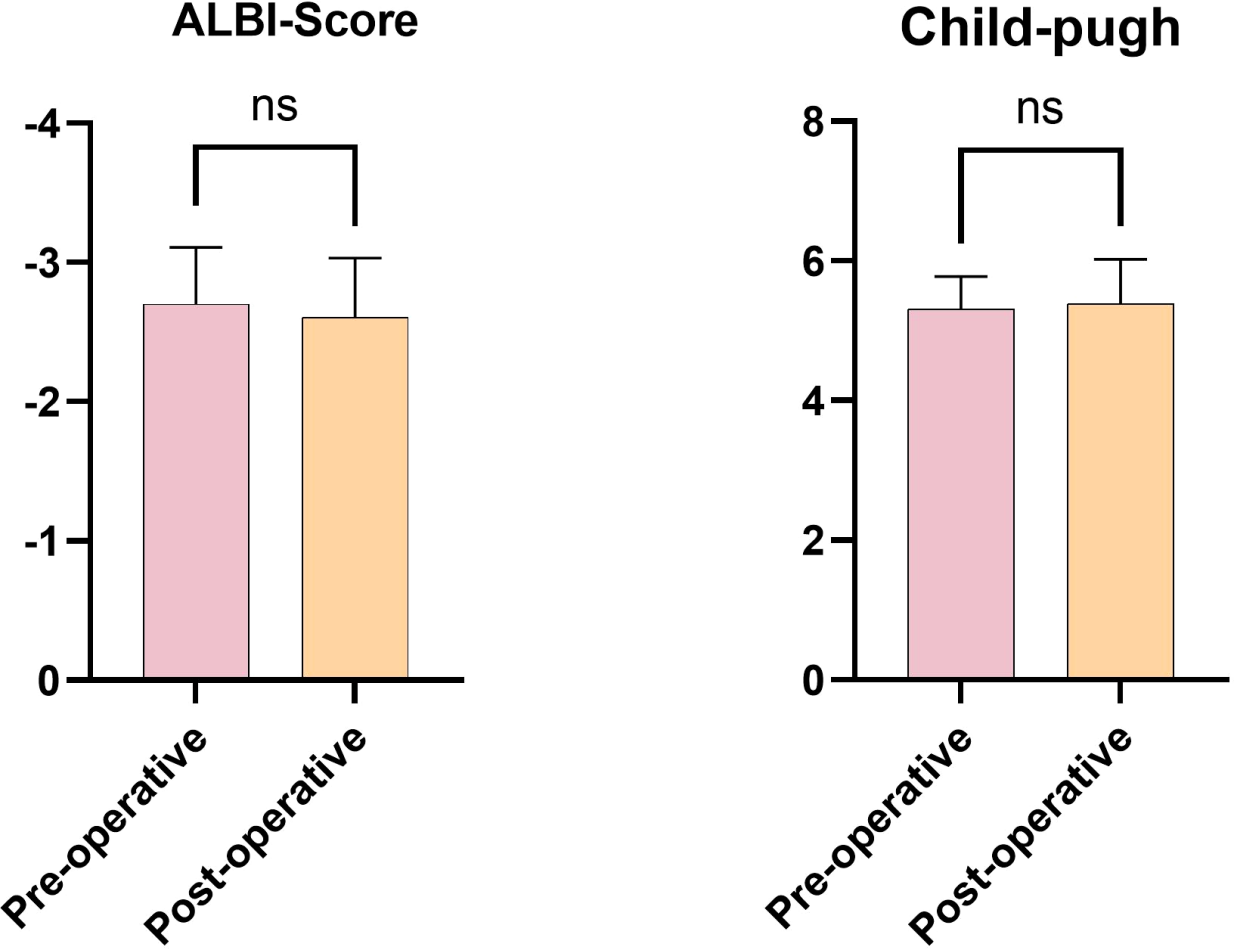
Correlation between APRI growth rate and FIB-4 growth rate. NS, Not significant.

The FIB-4 index was elevated in 42 patients (80.77%) after transarterial perfusion chemotherapy, including twelve patients with FIB-4 > 3.25; meanwhile, 42 patients (80.77%) had elevated APRI values post-procedure, with three having an APRI > 0.79. A strong correlation was observed between the magnitude of increase in FIB-4 and APRI, with a correlation coefficient of 0.93 (P < 0.05) (**Figure 4**). The hepatic fibrosis indices FIB-4 and APRI of patients after transarterial infusion chemotherapy were significantly greater than pre-treatment, with the differences being statistically significant after applying the Benjamini-Hochberg procedure for controlling the false discovery rate (adjusted P < 0.01) for multiple comparisons (**Table 3**).

**Table 3:** Hepatic fibrosis indices.

			*t*	*p*
	Pre-operative	Post-operative		
FIB-4	1.29 ± 0.88	2.24 ± 1.38	-5.07	<0.01**
APRI	0.19 ± 0.15	0.37 ± 0.27	-3.89	<0.01**

** *p*<0.01.

Changes in preoperative and postoperative hepatic fibrosis indices.

The paired t-test analysis indicated that postoperative FIB-4 and APRI scores were significantly higher than preoperative scores (P<0.01), with a statistically significant difference.

### 3.5 Liver function indicators

**Figure 5:**
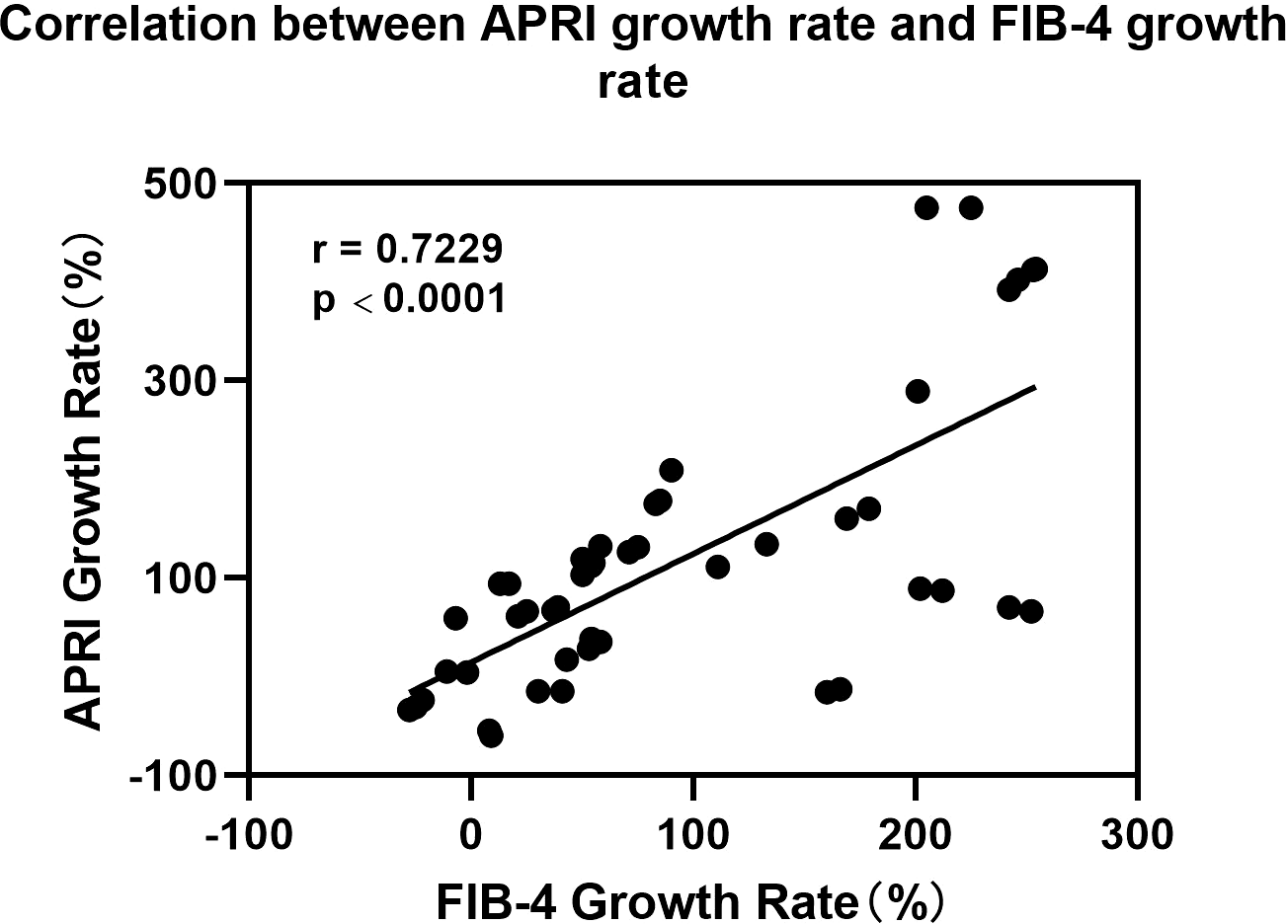
Changes in preoperative and postoperative liver function scores.

Changes in liver function indicators before and after treatment, as assessed using the drug indices of liver damage (including glutamic aminotransferase, glutamic alanine aminotransferase, total bilirubin, and serum albumin), were not statistically significant after applying the Benjamini-Hochberg procedure for controlling the false discovery rate (adjusted P > 0.05) (**Figure 5**).

## 4 Discussion

In recent years, several studies have highlighted the promising clinical outcomes of transarterial infusion chemotherapy in the treatment of progressive gastric cancer. The treatment regimen used in this study was based on a previous investigation by Li et al. (23–25). The arterial chemotherapeutic agents used included oxaliplatin, etoposide, and epirubicin, combined with oral S-1 chemotherapy. Although this treatment regimen has demonstrated promising therapeutic efficacy in clinical settings, there are limited reports on its potential side effects, particularly damage to adjacent organs, such as the liver. In recent years, with the advancement of medical imaging technology, radiomics, particularly quantitative imaging analysis, has become increasingly widespread. As an effective evaluation method, quantitative imaging analysis has been extensively applied in the clinical monitoring of functional and structural changes in the liver and spleen (26, 27). Changes in the L/S density ratio have been shown to be significantly correlated with chemotherapy-induced liver injury, while an increase in splenic volume is closely associated with the degree of systemic inflammatory response and liver damage. Therefore, in this study, based on imaging parameters such as the L/S density ratio and splenic volume, in combination with corresponding hepatic fibrosis markers, we identified latent liver damage caused to the normal hepatic parenchyma during chemotherapy in the progression of advanced gastric cancer.

The L/S density ratio is currently the main noninvasive method for diagnosing fatty liver and assessing the presence of steatosis in donor livers prior to liver transplantation. The diagnosis of fatty liver is based on the principle that fat accumulation in the liver leads to decreased liver density and lower CT values. A liver density lower than that of the spleen indicates higher diagnostic specificity, with an L/S density ratio of <1 being the recognized criterion. As this ratio decreases, the severity of fatty liver worsens (28, 29). Hepatocellular steatosis is a key indicator for assessing the quality of living donor livers before liver transplantation, with optimal donors having steatosis below moderate levels (< 30%). Thus, noninvasive and quantitative assessment of hepatic steatosis ≥ 30% has become a research focus. In previous studies on CT*_L/S_* (the liver-to-spleen attenuation ratio. It was calculated as L/S, where L is the hepatic attenuation and S is the splenic attenuation) in the quantitative diagnosis of hepatic steatosis (29, 30), the specificity and sensitivity of the diagnosis of hepatic steatosis >30% were 100% and 82%, 97% and 79%, and 83% and 82% when the critical values of L/S density ratio were set to 0.80, 0.90, and 1.10, respectively. Hence, the L/S density ratio is an important method for noninvasively quantifying hepatic steatosis, helping to avoid unnecessary liver biopsies. Previous studies have reported chemotherapy-associated liver injury, with pathologic changes including hepatocellular steatosis (31–33). For example, Vauthey et al. found that a 17.32% incidence of moderate-to-severe hepatic steatosis-like changes in patients with colorectal cancer liver metastases who received preoperative intravenous chemotherapy (34). Additionally, Paul Ryan et al. (35) reported that 9.90% of 334 patients with liver recurrence after colorectal cancer surgery had significant steatosis (>33%) following intravenous adjuvant chemotherapy prior to hepatectomy. A limitation of these studies is that all patients had liver lesions pre-surgery, and although the pathology samples were taken from non-tumor liver tissue, baseline characteristics varied. To further investigate the impact of chemotherapeutic agents on non-tumor liver parenchyma, our study selected patients with advanced gastric cancer who had normal liver function before surgery. We utilized a noninvasive method (L/S density ratio) to assess liver parenchyma damage and evaluate the presence of steatosis or steatohepatitis (36, 37). In this study, the postoperative L/S density ratio of the patients was significantly lower than that in the preoperative period (P<0.05). A study conducted by Kanae Miyake and colleagues investigated the effects of oral 5-fluorouracil (5-FU) therapy on liver fat content in patients with colorectal cancer. The findings revealed a significant reduction in liver CT values following adjuvant chemotherapy with oral 5-FU. We have shown a statistically significant decrease in hepatic CT values associated with adjuvant chemotherapy using oral 5-FU drugs in patients with colon cancer, which was observed even without abnormalities in laboratory data (38).Our findings align with these results, suggesting that the observed reduction in postoperative liver CT attenuation values is attributable to the oral administration of S-1. This reduction indicates an increase in liver fat content, which may contribute to the frequent occurrence of hepatic steatosis in these patients. Tegio is a fluorouracil derivative that is usually administered orally. The oral administration of 5-FU analogs has been associated with increased hepatic fat content in patients. A kinetic study employing magnetic resonance spectroscopy (MRS) revealed that during intra-arterial or intravenous infusion of 5-FU, the drug itself cleared from circulation relatively quickly. However, FBAL( (Alpha-fluoro-beta-alanine)), a major metabolite of 5-FU, gradually accumulated in the liver, leading to an increase in liver fat content in affected patients (39).Potential mechanisms underlying the increase in liver fat content may include: 1. Exposure to FBAL, a fluorinated compound, which induces significant alterations in lipid-related metabolites, including lysophosphatidylethanolamine and lysophosphatidylcholine, thereby impacting hepatic lipid metabolism and contributing to an elevation in hepatic fat content (40); 2. The accumulation of FBAL may disrupt mitochondrial membrane integrity and decrease membrane potential, impairing mitochondrial function. These effects can contribute to liver injury by interfering with key cellular processes and energy production (41).

In this study, patients showed a significant increase in spleen volume postoperatively (P<0.05), which we consider to be another manifestation of hepatic damage induced by chemotherapeutic agents infused through the transcatheter trunk artery. A prospective study enrolling 50 patients with malignant tumors receiving intravenous chemotherapy containing oxaliplatin showed that 37 patients (74%) had an increase in splenic volume after chemotherapy, with a median increase of 31% and a maximum increase of 119% at 3 months (42). In another retrospective study of 161 patients receiving oxaliplatin-containing intravenous chemotherapy, CT imaging assessed spleen size after 6 months of treatment. An increase in splenic volume was observed in 104 patients (64.60%), with 19.43% of the patients having an increase of ≥30%, and 8.10% having an increase of ≥50% (43). Toi H et al. (44) investigated the relationship between the FOLFOX regimen and spleen volume, showing that after 6 months of FOLFOX intravenous chemotherapy, spleen volume (measured on CT images) increased from 229 cm³ to 323 cm³ (P<0.01), with 44.41% of patients showing an increase of >50%. The increase in splenic volume and hepatic sinusoidal injury due to chemotherapeutic agents, especially oxaliplatin, are closely related. Rubbia-Brandt et al. (33) retrospectively analyzed 153 patients with liver metastases from colorectal cancer; 44 of 87 patients (50.57%) who received preoperative oxaliplatin-containing chemotherapy developed pathological changes of hepatic sinusoidal damage, whereas the 66 patients who did not receive preoperative chemotherapy presented with normal livers. This study noted that liver pathology after surgical resection in patients receiving preoperative intravenous chemotherapy with oxaliplatin showed hepatic sinusoidal damage. Manifestations included sinusoidal dilatation, hemorrhage, thickening of small hepatic veins, fibrosis, and luminal narrowing or occlusion, occurring in 51%-78% of cases. Another study found that in patients with liver metastases treated preoperatively with 5-FU analogues, fluoropyrimidine, and oxaliplatin, both univariate (P=0.03) and multivariate (P=0.02) analyses showed increased spleen volume was associated with hepatic sinusoidal injury and was an independent predictor of severe injury (45). Oxaliplatin chemotherapy is associated with histologic changes in the liver characterized by dilated hepatic sinusoids, congestion, and centrilobular necrosis suggestive of hepatic sinusoidal obstruction syndrome. These changes are not clinically significant in the acute phase and are not accompanied by markedly elevated serum enzyme profiles or clinically significant liver injury (46). Our study is consistent with the findings of this experiment. However, long-term administration of oxaliplatin results in endothelial damage to the hepatic sinusoids within the liver tissue, which leads to non-cirrhotic portal hypertension(NCPH). This condition arises from obstruction of the hepatic sinusoids and the subsequent development of nodular regenerative hyperplasia(NRH). NCPH typically progresses gradually and remains asymptomatic for an extended period until complications such as variceal bleeding and splenomegaly manifest (47). The developmental time for such changes is usually 6 to 18 months (46). In this study, we observed radiological evidence of splenomegaly as early as 2-3 cycles (with an average of 6.8 weeks) post-treatment. We hypothesize that this may be attributed to regional portal vein underperfusion following the intra-arterial infusion of high concentrations of chemotherapeutic agents via the coeliac trunk and hepatic artery. Insufficient blood perfusion induces hepatocyte atrophy and apoptosis, while the perfusion of surrounding acinar cells increases. In areas with excessive perfusion, there is an elevation of cell growth activators, which, acting as autocrine or paracrine peptides, contribute to the formation of NRH. This process leads to the development of splenomegaly following the onset of secondary NCPH (48).

To reduce drug-induced liver injury, the most crucial approach is to minimize the dosage of the drug used. To ensure that the tumor site reaches the same drug concentration, intra-arterial infusion, compared to intravenous administration, can reduce the drug dosage. More prospective studies should be conducted to further optimize the use of drug doses. For example, reducing the duration of drug infusion (49), or using balloon catheters to lower the arterial infusion pressure, can slow down the blood flow rate, thereby reducing the drug dosage while maintaining the same drug concentration and infusion time. In patients with metastatic colorectal cancer who received oxaliplatin-based chemotherapy combined with bevacizumab, a longer median time to splenomegaly and a lower incidence of thrombocytopenia were observed (50). These approaches may delay liver function damage.

The histological manifestations associated with drug-induced liver injury are complex and varied, and the progression can be as severe as that of hepatic fibrosis and cirrhosis (51). FIB-4 and APRI indices are correlated and consistent with the degree of hepatic fibrosis and are commonly used to noninvasively assess the degree of hepatic fibrosis and cirrhosis in patients with chronic viral hepatitis. Rubbia-Brandt et al. (33) reported that 48% of liver pathological tissues obtained after intravenous chemotherapy exhibited steatosis and hepatic fibrosis. Therefore, to noninvasively assess chemotherapy-related hepatic fibrosis, indicators such as FIB-4, APRI, and ALBI have attracted the attention of researchers (52). In a study by Mukai et al. (43), the median FIB-4 index was significantly higher after 6 months of intravenous chemotherapy (1.28 vs 2.50, P<0.01), and nine of these patients developed portal hypertension with portal collateral circulation formation; the authors concluded that the FIB-4 index can be used as a useful predictor of portal hypertension with portal collateral circulation formation. The results of a study by Xuelin et al. on liver injury associated with intravenous chemotherapy for colorectal cancer showed a good correlation between the increase in FIB-4 and APRI (P<0.05) and a positive correlation between the increase in spleen volume and the increase in FIB-4 and APRI (P<0.01), with correlation coefficients of 0.36 and 0.58, respectively (53). Combined with related studies, we believe that FIB-4 and APRI can be used as noninvasive biological indices to monitor tumor chemotherapy-related liver injury and can reflect early hepatic sinusoidal injury more sensitively. In this study, the FIB-4 and APRI indices before and after transarterial infusion chemotherapy were significantly higher (P<0.01), suggesting the development of hepatic fibrosis in the short term after transarterial infusion chemotherapy. In conjunction with related studies, Gu et al. (54)observed that epothilone-treated FGF1 knockout mice exhibited worsening of intestinal fibrosis, accompanied by increased upregulation of connective tissue growth factor expression, a well-established marker of fibrosis. Similarly, a study by I.L. Ke Nalbantoglu et al. reported that the livers of patients treated with oxaliplatin chemotherapy demonstrated capillarization, hepatic sinusoidal fibrosis, and hepatocellular hyperplasia following hepatectomy (55). Based on these findings, we hypothesize that the liver fibrosis observed may result from the combined effects of oxaliplatin and epothilone. However, the exact underlying mechanism remains unclear, and further animal experiments will be conducted to investigate the mechanism of injury in greater detail.

According to the literature (56), chemotherapy-associated liver injury is routinely detected and diagnosed using laboratory tests for liver function, which typically include elevated ALT, ALP, and bilirubin levels, decreased albumin levels, or poor coagulation. In this study, the preoperative and postoperative laboratory indices of liver function, which were within the normal range, failed to meet the common diagnostic criteria for drug-induced liver injury. Simultaneously, there was no significant difference between the ALBI and traditional Child-Pugh scores pre- and post-operatively. Therefore, we believe that liver damage from transarterial infusion chemotherapy is predominantly chronic, and this insidious pharmacological liver damage can be detected by comparing preoperative and postoperative L/S density ratio, spleen volume, and noninvasive hepatic fibrosis assessment indices (FIB-4, APRI). Serum enzyme profiles, ALBI scores, and Child-Pugh scores are not sensitive to this liver injury, particularly in the early stages.

Our study had several major shortcomings. Firstly, this was a single-center, retrospective study that included a small sample size. And there were too many subsequent treatment factors to allow for further follow-up, resulting in a shorter follow-up period. Longer follow-up periods will provide a more complete assessment of the long-term effects of treatment on the liver. Secondly, the chemotherapy drugs selected in this study are rarely used alone in clinical practice but rather administered in combination. Therefore, the mechanism of liver injury caused by these drugs are complex, and it is difficult to determine whether a particular drug causes the abovementioned liver injury. Because the patients in this study had advanced gastric cancer and normal liver function before surgery, we could not obtain ethical approval for liver biopsy, limiting out ability to acquire definitive pathological tissues and clarify the type of liver damage. To address these limitations, further animal studies will be conducted to determine which drug caused liver damage and the nature of that damage.

## 5 Conclusion

Transarterial infusion chemotherapy can lead to subtle liver damage within a relatively short period, including hepatic steatosis, hepatic sinusoidal obstruction-like changes, and fibrosis. CT imaging and hepatic injury indicators may detect drug-induced hepatic damage before laboratory abnormalities or clinical manifestations become apparent. It is crucial for interventional radiologists to be aware of this potential side effect when monitoring patients undergoing transarterial infusion chemotherapy, as they may be the first to identify it and optimize therapeutic management.

## Data Availability

The raw data supporting the conclusions of this article will be made available by the authors, without undue reservation.
